# Complete mitochondrial genome sequence of *Gymnopilus junonius*

**DOI:** 10.1080/23802359.2021.1895692

**Published:** 2021-03-18

**Authors:** Sung Eun Cho, Jong Won Jo, Young-Nam Kwag, Hyun Lee, Jong-Wook Chung, Seung Hwan Oh, Chang Sun Kim

**Affiliations:** a Forest Biodiversity Division, Korea National Arboretum, Pocheon, South Korea; b Department of Industrial Plant Science and Technology, Chungbuk National University, South Korea

**Keywords:** Basidiomycota, Hymenogastraceae, phylogenetic analysis, poisonous mushroom

## Abstract

*Gymnopilus junonius*, a well-known poisonous mushroom, is distributed worldwide. It contains a hallucinogenic alkaloid psilocybin and several other bioactive compounds. The mitochondrial genome, a circular DNA molecule of 161,145 bp, comprises 15 protein-coding genes, 24 transfer RNA genes, and 2 ribosomal RNA genes. The guanine-cytosine content was 31.56%. Based on the mitochondrial genome sequence, a phylogenetic tree was constructed to demonstrate the phylogenetic relationship. In this study, the phylogenetic positions of *G*. *junonius* and its related genera were determined.

*Gymnopilus junonius* (Fr.) P.D. Orton is a well-known poisonous mushroom and contains psilocybin, a hallucinogenic alkaloid that is chemically related to the amino acid tryptophan (Chilton et al. 1979). Psilocybin is present in over 200 species of Basidiomycota mushrooms, including *Gymnopilus* (Guzmán et al. 1998). In addition, several bioactive compounds, such as gymnopilins, cerevisterol, acetylenic compounds, trichothecene, and tremulane sesquiterpenes, have been reported in *Gymnopilus* sp. (Kim et al. 2012; Lee et al. 2008, 2020). Hence, *G. junonius* is medicinally valuable and serves as a good source for research on hallucinogenic mushrooms. In this study, the complete mitochondrial genome of *G. junonius* was reported to confirm the phylogenetic relationship between the related genera.

Samples of *G. junonius* were collected from Gwangneung Forest, Pocheon-si, Gyeonggi-do, South Korea (37°45′N, 127°10′E) and deposited at the herbarium of the Korea National Arboretum (KH; voucher no. KA18-0872C). Genomic DNA was then isolated from the mycelium and subjected to the construction of an Illumina paired-end (PE) library according to the manufacturers’ protocol. The library was sequenced using Illumina PE sequencing at the PHYZEN Genomics Institute (Phyzen, Gyeonggi-do, South Korea). High-quality PE reads obtained after trimming were *de novo* assembled using the CLC genome assembler (v. 4.21, CLC Inc., Denmark). From the initially assembled contigs, those derived from the mitochondrial genome sequences were further processed to generate a single draft sequence, as described previously (Lee et al. 2018). The draft sequence was manually corrected and gap-filled using a series of PE read mapping. The complete mitochondrial genome sequence was annotated using GeSeq (https://chlorobox.mpimp-golm.mpg.de/geseq-app.html) and manually curated using the Artemis annotation tool (Rutherford et al. 2000) with NCBI BLASTN searches.

The complete mitochondrial genome sequence of *G*. *junonius* (GenBank Accession no. MW238478) was 161,145 bp in length, second largest among the previously reported mitogenomes of Agaricales. It consisted of 15 protein-coding genes, 24 transfer RNA genes, and 2 ribosomal RNA genes. The base composition was adenine (34.42%), cytosine (C) (15.41%), guanine (G) (16.14%), and thymine (34.01%). Overall GC content of the mitochondrial genome was 31.56%.

Phylogenetic analysis was performed based on the multiple alignments of protein-coding sequences in mitochondrial genomes (Kumar et al. 2016). Results revealed that *Agrocybe aegerita* (MF979820) was placed in a sister clade. In this study, the phylogenetic position of genus *Gymnopilus* was reported for the first time (Figure 1). Information on the mitochondrial genome sequence of *Gymnopilus* sp. would help understand species evolution and phylogenetic relationships between the related taxa.

**Figure 1.**
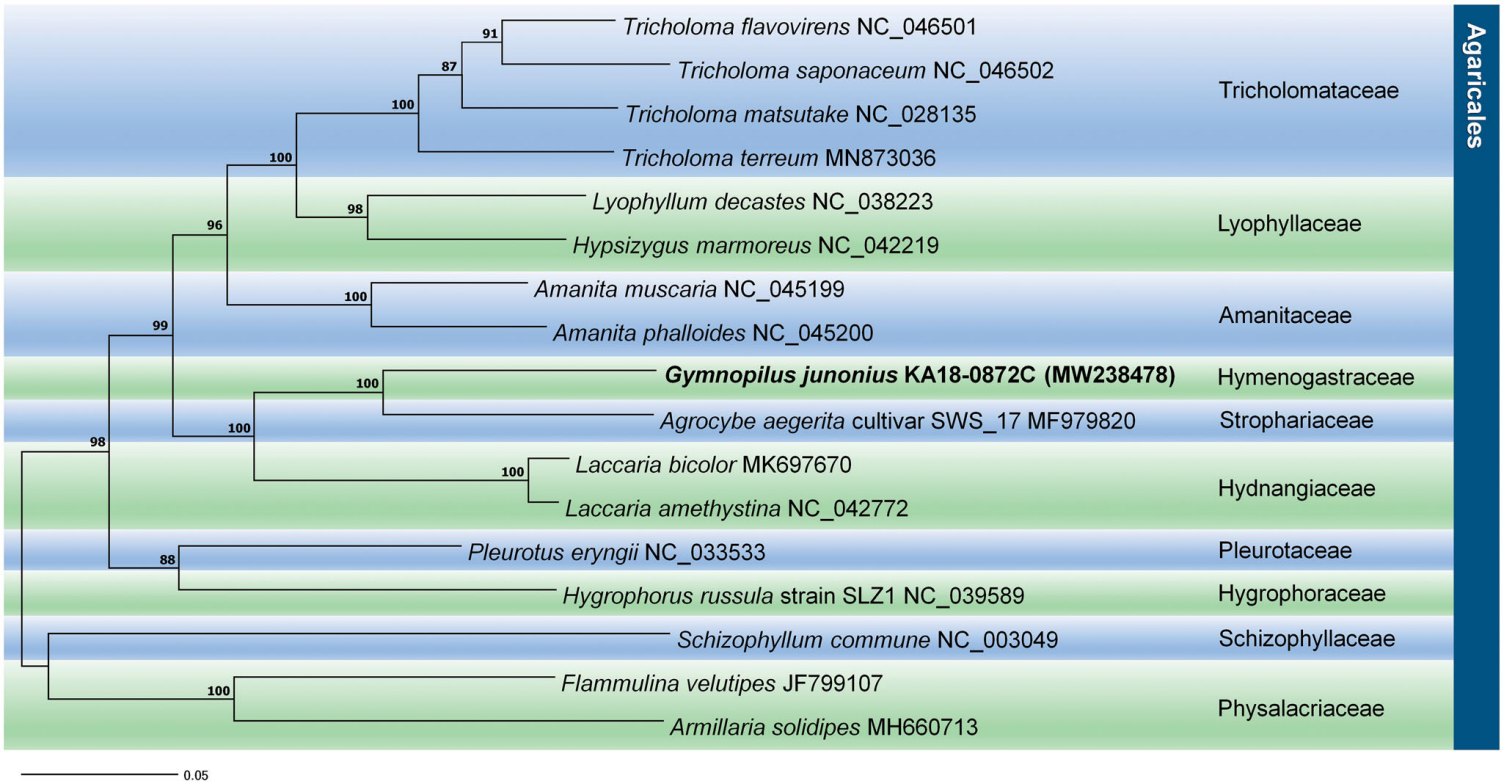
Phylogenetic tree of *Gymnopilus junonius* and 16 other related taxa. Sequences of protein-coding regions in the mitochondrial genome were aligned using MAFFT and MEGA 7.0. Numbers in the nodes indicate bootstrap support values (>80%) from 1000 replicates.

## Data Availability

The genome sequence data that support the findings of this study are openly available in GenBank of NCBI at (https://www.ncbi.nlm.nih.gov/) under the accession no. MW238478. The associated BioProject, SRA, and Bio-Sample numbers are PRJNA700711, SRX10055256, and SAMN17838281, respectively.
